# Milk Fat Content and DGAT1 Genotype Determine Lipid Composition of the Milk Fat Globule Membrane

**DOI:** 10.1371/journal.pone.0068707

**Published:** 2013-07-18

**Authors:** Nurit Argov-Argaman, Kfir Mida, Bat-Chen Cohen, Marleen Visker, Kasper Hettinga

**Affiliations:** 1 Department of Animal Sciences, The Robert H. Smith Faculty of Agriculture, Food and Environment, The Hebrew University of Jerusalem, Jerusalem, Israel; 2 Animal Breeding and Genomics Centre, Wageningen University, Wageningen, The Netherlands; 3 Dairy Science and Technology, Product Design and Quality Management Group, Wageningen University, Wageningen, The Netherlands; Wageningen UR Livestock Research, The Netherlands

## Abstract

During secretion of milk fat globules, triacylglycerol (TAG) droplets are enveloped by a phospholipid (PL) trilayer. Globule size has been found to be related to polar lipid composition and fat content, and milk fat content and fatty acid composition have been associated with the diacylglycerol acyltransferase 1 (DGAT1) K232A polymorphism; however, the association between the DGAT1 polymorphism and fat globule size and polar lipid composition has not been studied. The ratio between polar and neutral lipids as well as the composition of the polar lipids in milk has industrial as well as nutritional and health implications. Understanding phenotypic and genotypic factors influencing these parameters could contribute to improving milk lipid composition for dairy products. The focus of the present study was to determine the effect of both fat content and DGAT1 polymorphism on PL/TAG ratio, as a marker for milk fat globule size, and detailed PL composition. Milk samples were selected from 200 cows such that there were equal numbers of samples for the different fat contents as well as per DGAT1 genotype. Samples were analyzed for neutral and polar lipid concentration and composition. PL/TAG ratio was significantly associated with both fat content and DGAT1 genotype. Phosphatidylinositol and phosphatidylserine concentrations were associated with fat content*DGAT1 genotype with a stronger association for the AA than the KK genotype. Sphingomyelin concentration tended to interact with fat content*DGAT1 genotype. Phosphatidylethanolamine (PE) concentration showed a biphasic response to fat content, suggesting that multiple biological processes influence its concentration. These results provide a new direction for controlling polar lipid concentration and composition in milk through selective breeding of cows.

## Introduction

Dietary fat has been the focus of many health-related studies due to the connection between its consumption and health conditions such as obesity, diabetes and atherosclerosis. An increasing body of evidence indicates that it is not only the fat content of the diet but also its composition that should be considered, because of the difference in metabolic and health impact of different fatty acids and lipid species [1]–[3]. Fatty acids in the diet are almost never consumed as free fatty acids. Rather, they are present in foods as part of larger lipid molecules, primarily triacylglycerols (TAG) and, to much lesser extent, as polar lipids, glycerophospholipids and sphingolipids (i.e. phospholipids, PL). Consumption of diets rich in PL or TAG differ in metabolic outcome, suggesting a beneficial effect of a PL-rich diet [4]–[6]. Therefore, understanding the mechanisms determining the ratio between PL and TAG in food is of great importance.

Milk is one of the major sources of fat in the western diet [7]. The wide range of lipid species in milk is attributed to the unique fat-secretion pathway employed by the mammary gland [8]. Milk fat is secreted in a unique structure termed the milk fat globule (MFG) which consists of a TAG core covered with three layers of PL and proteins [9]. Milk fat consists of 96 to 97% TAG and 0.5 to 1% structural lipids, mainly PL that envelopes the TAG droplet during secretion [10], forming the milk fat globule membrane (MFGM) [11].

MFG are secreted in a wide range of sizes, with a diameter ranging from the nanometer scale to more than 15 µm [12]. Due to its unique structure, the MFG’s size determines the ratio between the lipids in its TAG core and its PL envelope [13], [14] and, thus, may be used to modulate and improve milk fat composition.

Amount and composition of milk fat depend on several factors, including animal characteristics such as breed, lactation stage and genetic polymorphisms (e.g. [16], [17]), and characteristics of the animal’s diet such as energy content and fat composition [8],[15]. In addition, a strong association between milk fat content and MFG size has been demonstrated in dairy cows [18], by diet-induced alterations in milk fat content [13], and by interspecies comparison of milk fat concentrations [19]. Milk fat content has also been associated with the composition of PL in MFG of dairy goats and cows [20], [21]. Furthermore, association between TAG and PL composition [12], [13], [14], [22] and MFG size has been illustrated in human as well as bovine milk. These data imply that the lipogenic capacity of the mammary gland, as reflected by milk fat content, is associated with MFG size, the composition of its PL envelope and the ratio between PL and TAG.

In the mammary gland, the primary product of the lipogenic process is TAG. The last stage in TAG synthesis is catalyzed by the enzyme diacylglycerol acyl transferase 1 (DGAT1) [23]. A genetic polymorphism (K232A) in the gene encoding the enzyme DGAT1 has been shown to have an effect on milk fat content [16]. The DGAT1 K allele is associated with increased milk fat content, which has been attributed to increased V*max* of the K variant of the enzyme [16]. The increased V*max* of the K variant can result in a changed composition of the DAG pool [17] which may affect both neutral and polar lipid composition in the cell. Whether the DGAT1 polymorphism is associated with the distribution of milk fat between PL and TAG, and whether this is reflected in the composition of the MFGM and PL/TAG ratio in milk, has never been studied.

In this study we determined the effect of both fat content and DGAT1 K232A polymorphism on PL/TAG ratio and detailed PL composition. The results should lead to a better understanding of factors determining MFG size, MFGM composition, and the milk fat secretion process in mammary epithelial cells.

## Materials and Methods

### Sample Selection

Phospholipid content and composition was determined in samples of raw morning milk taken in winter from 204 first-lactation Dutch Holstein Friesian cows. These samples were a selected subset of the winter milk samples that were taken from 2,000 cows for the Dutch Milk Genomics Initiative. The 204 cows were housed on 160 farms throughout the Netherlands and were between 67 and 263 days in milk at the time of sampling.

For milk samples of all 2,000 cows, fat content was measured by infrared spectroscopy using a MilkoScan FT6000 (Foss Electric, Hillerod, Denmark) at the Milk Control Station (nowadays Qlip NV, Zutphen, the Netherlands). Genomic DNA was isolated from whole blood samples of all cows and genotyped for the DGAT1 K232A polymorphism with a Taqman allelic discrimination assay, as described by [17]. Blood samples were collected in accordance with the guidelines for the care and use of animals as approved by the ethical committee on animal experiments of Wageningen University (protocol: 200523.b).

The subset of 204 samples was selected based on DGAT1 genotype and fat content. About half of the samples (100) were from cows with the DGAT1 AA genotype and half of the samples (104) were from cows with the DGAT1 KK genotype. Samples within each genotype represented the variation in fat percentages that was present in the samples of all 2,000 cows (between 2.5 and 7.5%). In addition, samples that had phenotypic values of more than 2 standard deviations from the mean for selected traits (milk yield, protein content, lactose content, somatic cell count, and the fatty acids C4∶0, C6∶0, C8∶0, C10∶0, C12∶0, C14∶0, C16∶0, C18∶0, C18∶1cis9, C:18∶2cis9,12; determined as described by [17] and [24]) were not selected.

### Lipid Extraction and Analysis

#### Chemicals and reagents

For lipid extraction, analytical reagent-grade methanol and chloroform were purchased from Bio-Lab Ltd. Laboratories (Jerusalem, Israel). For HPLC analysis, dichloromethane, methanol and ethanol, HPLC-grade and analytical reagent-grade, were purchased from Bio-Lab. The triglyceride standard triolein (>99% pure) was purchased from Supelco (Bellefonte, PA, USA). Cholesterol (>99% pure) and PL standards were supplied by Sigma Aldrich Israel Ltd. (Rehovot, Israel), and consisted of phosphatidylethanolamine (PE) (1,2-dioleoyl-sn-glycero-3-phosphoethanolamine, purity 99%), phosphatidyl inositol (PI) (L-α phosphatidylinositol ammonium salt, from bovine liver, purity 98%), phosphatidylserine (PS) (1,2-dioleoyl-sn-glycerol-3-phospho-L-serine sodium salt, purity 95%), phosphatidylcholine (PC) (1,2-dioleoyl-sn-glycero-3-phosphocholine, purity 99%), sphingomyelin (SM) (from bovine brain, purity 97%). As an internal standard for free fatty acids, C11∶0 (undecanoic acid, purity 99%) from Sigma Aldrich was used.

#### Extraction of total lipids from milk

A protocol adapted from the cold extraction procedure developed by Folch et al. [25] was used for the extraction of total lipids from the milk. Total lipids were extracted from 0.5 mL milk with 10 mL chloroform-methanol (2∶1, vol/vol) as described previously [21]. For the HPLC analysis, 100 µL chloroform–ethanol (97∶3 vol/vol) was added to the evaporated tubes containing lipids and stored at -20°C until injection into the HPLC.

#### HPLC analysis of PL and TAG

Quantification of PL and TAG and determination of lipid class were performed by HPLC (HP 1200, Agilent Technologies) combined with an evaporative light-scattering detector (ELSD1200, Agilent Technologies). The separation process was managed by ChemStation software (Agilent Technologies), which permitted the acquisition of data from the ELSD detector, with an injection volume of 10 µL. The separation protocol was conducted as previously described by Argov-Argaman et al. [20] using normal-phase chromatography on a silica column (Zorbax, Agilent Technologies). Calibration and lipid concentration and composition were determined using external standards (Sigma Aldrich).

#### HPLC/ELSD calibration

PL were identified and quantified by normal-phase liquid chromatography (HP 1200, Agilent Technologies) equipped with ELSD (1200 series ELSD, Agilent Technologies). The method employed for lipid separation, consisting of dichloromethane, methanol and double-distilled water, was as previously described [14]. Briefly, a column (Zorbax RX-SIL, 4.6×250 mm, Agilent Technologies) was heated to 50°C, and flow was set to 1 mL/min. The ELSD was heated to 65°C, nitrogen pressure was 3.9 bar, a no. 5 filter was used, and gain (sensitivity) was set to 7 for the first 11 min and then changed to 9 until the end of the run to enable detection of lower-abundance lipid components. Injection volume was 20 µL. This protocol induced the separation of TAG, two isomers of diacylglycerol, monoacylglycerol, cholesterol, free fatty acids, PE, PI, PS, PC and SM. Quantification was based on areas under the standard curves of each lipid standard concentration. The power equations were: triglyceride, y = 0.0014x^0.8695^ (r^2^ = 0.995); cholesterol, y = 0.0245x^0.581^ (r^2^ = 0.9925); PE, y = 0.1369x^0.437^ (r^2^ = 0.9908); PI, y = 0.0103x^0.7918^ (r^2^ = 0.9898); PS = 1.73X^0.41^ (r^2^ = 0.99), PC, y = 0.0408x^0.5077^ (r^2^ = 0.9986), and SM, y = 0.0667x^0.5287^ (r^2^ = 0.9981).

### Statistical Analysis

Data analysis was performed first using SAS 9.2 (SAS Institute Inc., Cary, USA) to determine fixed effects. Subsequently, data were analysed using the following animal model in ASReml [26]:
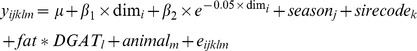
where *y_ijklm_* is an observation of animal *m*, with fat content*DGAT1 genotype interaction (fat*DGAT1) *l*, sirecode *k*, season of calving (season) *j*, and days in milk (dim) *i*; *µ* is the general mean; dim*_i_* is a covariate for the effect of days in milk, modelled with a Wilmink curve [27]; season*_j_* is a fixed effect with 3 classes for season of calving, summer (June to August 2004), autumn (September to November 2004), and winter (December 2004 to February 2005); sirecode*_k_* is a fixed effect accounting for possible differences in genetic level between the groups of proven bull daughters and young bull daughters; fat*DGATl is a fixed effect for the interaction between fat content and DGAT1 polymorphism with two classes for DGAT1 genotype (AA and KK); animal*_m_* is a random additive genetic effect for animal, based on a pedigree of 26,300 animals; and e*_ijklm_* is a random residual effect. Including only AA and KK genotypes in the analysis, resulted in reporting only additive effects of the DGAT1 polymorphism.

The animal model uses heritability estimates that are relatively unreliable, because estimates are based on only 204 observations. To test whether this affected the results, all analyses were repeated with a fixed heritability of 0.1 and with a fixed heritability of 0.4. These analyses showed that the heritability hardly affected the test-statistics or the effects of significant associations, thus, that the relatively unreliable heritability estimates did not have a large impact on the analyses.

## Results

### Phospholipid Content and Composition

The composition of the total lipid fraction isolated from the 204 milk samples was determined by HPLC. Table 1 shows data for fat content, TAG content, PL content and relative concentrations of individual PL for KK and AA genotypes. Milk fat consisted of approximately 98% TAG and 1% PL, resulting in an average PL/TAG ratio of 0.01, with values ranging from 0.0095 to 0.0176 for the AA groups and from 0.0076 to 0.023 for the KK group. The individual phospholipids ranged in average concentration from 3.8% for PE in the KK group to 36.7% for PC in the AA group.

**Table 1:** Range, mean and SD of lipid extract composition from 204 milk samples.

	DGAT1 KK				DGAT1 AA			
	Min	Mean	Max	SD	Min	Mean	Max	SD
Fat content (%)	3.55	4.95	7.42	0.82	2.25	3.99	5.71	0.81
PL (% of TL)	0.63	0.94	1.72	0.21	0.75	1.22	2.24	0.30
TAG (% of TL)	97.60	98.52	99.01	0.27	96.77	98.14	98.81	0.44
TC (% of TL)	0.35	0.57	0.88	0.13	0.41	0.68	1.12	0.17
PL/TAG ratio	0.006	0.009	0.0176	0.002	0.0076	0.0124	0.0231	0.0032
PI (% of TPL)	7.11	17.11	28.56	4.59	8.45	17.64	27.71	4.32
PE (% of TPL)	3.89	8.69	15.88	2.67	3.83	9.10	16.73	2.52
PS (% of TPL)	10.62	19.35	32.00	4.60	11.75	19.73	36.33	5.46
PC (% of TPL)	24.73	30.06	36.20	2.28	23.30	29.99	36.78	2.29
SM (% of TPL)	18.29	24.80	38.34	3.60	9.47	23.54	31.07	3.16

PL: phospholipids; TAG: triacylglycerols; TL: total lipids; TC: total cholesterol; TPL: total phospholipids; PI: phosphatidylinositol; PE: phosphotidylethanolamine; PS: phosphatidylserine; PC: phosphotidylcholine; SM: sphingomyelin.

### Effect of Fat Content and DGAT1 Genotype on TAG Content and PL Content and Composition

**Figure 1:**
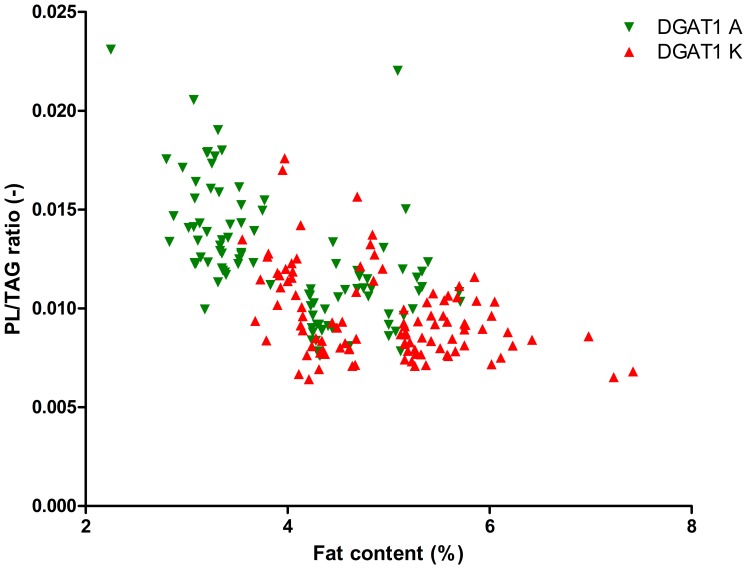
Fat content (%) versus phospholipid/triacylglycerol (PL/TAG) ratio (−) differentiated by DGAT1 genotype.

**Figure 2:**
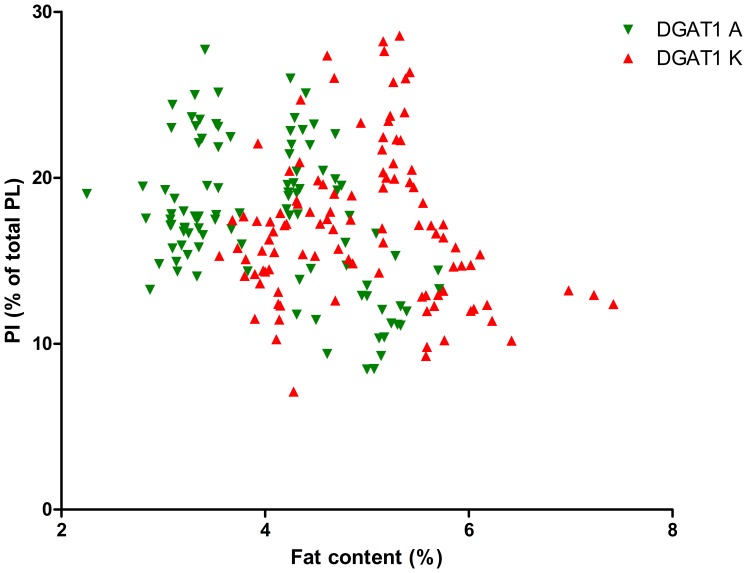
Relationship between fat content (%) and phosphatidylinositol (PI) content (% of total phospholipids (PL)) differentiated by DGAT1 genotype.

**Figure 3:**
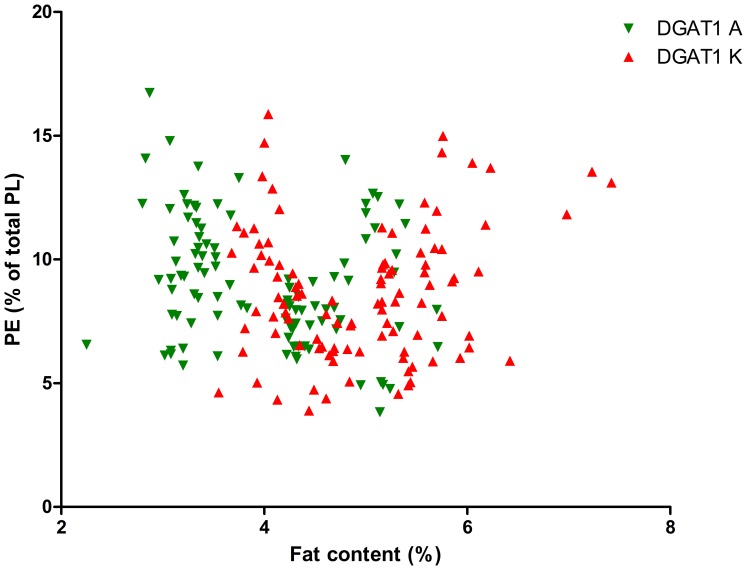
Relationship between fat content (%) and phosphotidylethanolamine (PE) content (% of total phospholipids (PL)) differentiated by DGAT1 genotype.

**Figure 4:**
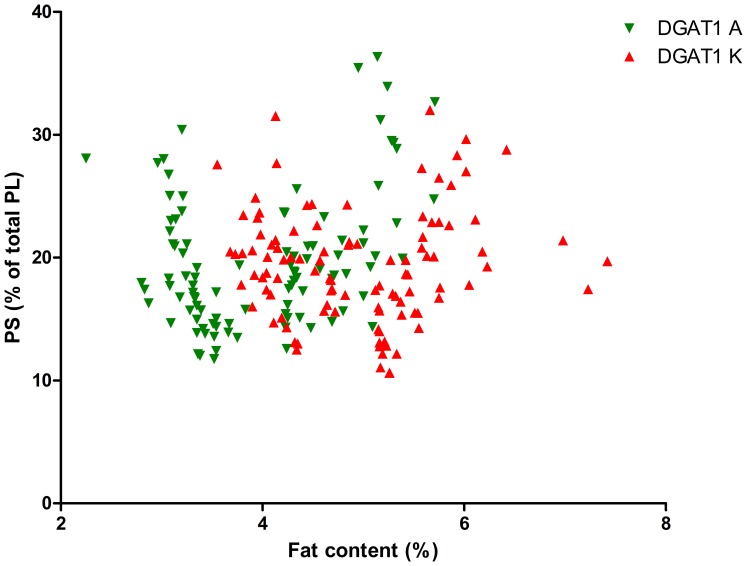
Relationship between fat content (%) and phosphatidylserine (PS) content (% of total phospholipids (PL)) differentiated by DGAT1 genotype.

**Figure 5:**
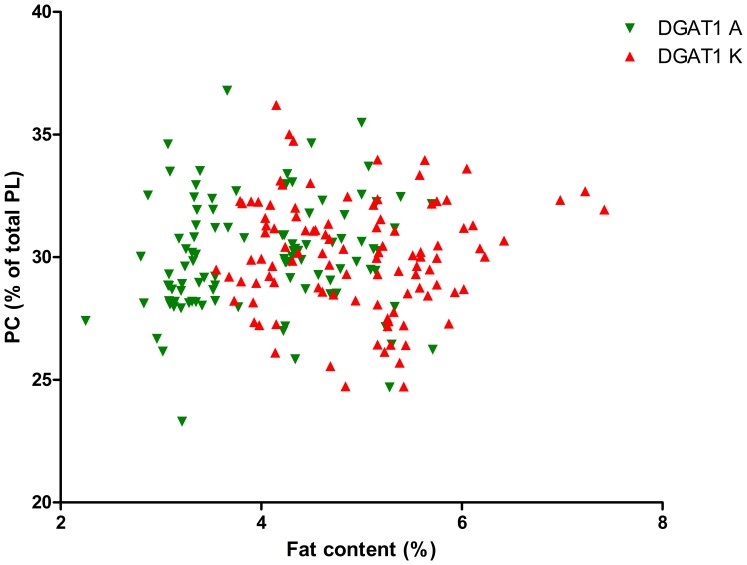
Relationship between fat content (%) and phosphatidylcholine (PC) content (% of total phospholipids (PL)) differentiated by DGAT1 genotype.

**Figure 6:**
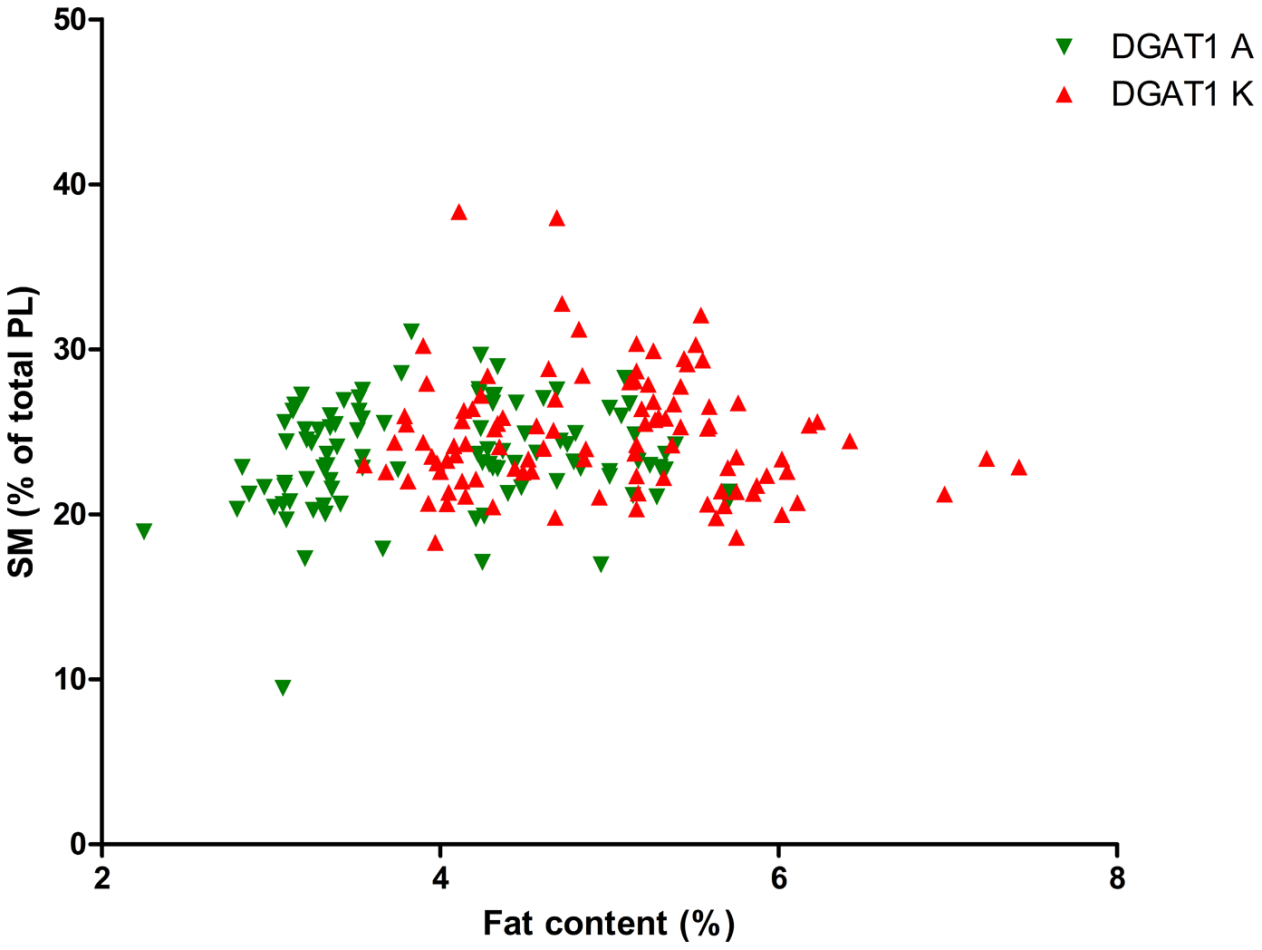
Relationship between fat content (%) and sphingomyelin (SM) content (% of total phospholipids (PL)) differentiated by DGAT1 genotype.

As the DGAT1 genotype is strongly correlated with fat content, we calculated the significance of the interaction between fat content and DGAT1 genotype on TAG content and on PL content and composition, see table 2. This table shows that the fat content*DGAT1 genotype interaction was significant for the PL/TAG ratio and for PI, PS and SM (P<0.1). Figure 1 shows the relation between Pl/TAG ratio and fat content and Figures 2–6 show the relation between the individual phospholipids and fat content, differentiated by DGAT1 genotype. The decrease in PL/TAG ratio with increasing fat content (Figure 1) and the increase in SM with increasing fat content (Figure 6) were both larger for the DGAT1 KK genotype than for the AA genotype. The decrease in PI with increasing fat content and the increase in PS with increasing fat content were both larger for the DGAT1 AA genotype compared with the KK genotype.

**Table 2:** Effect of the interaction between fat content and DGAT1 genotype on triacylglycerol content and on phospholipid content and composition.

	Fat*DGAT1	Fat*DGAT1 KK		Fat*DGAT1 AA	
Trait	*P*-value	Regressioncoefficient	SE	RegressionCoefficient	SE
PL (% of TL)	1.65*10^−20^	−0.177	0.0193	−0.155	0.0238
TAG (% of TL)	1.38*10^−21^	0.258	0.0262	0.236	0.0324
TC (% of TL)	4.06*10^−12^	−0.085	0.0115	−0.085	0.0142
PL/TAG ratio	1.82*10^−20^	−0.002	0.0002	−0.002	0.0002
PI (% of TPL)	0.001	−1.324	0.3596	−1.605	0.4448
PE (% of TPL)	0.638	−0.201	0.2178	−0.210	0.2681
PS (% of TPL)	0.013	0.995	0.4114	1.470	0.5092
PC (% of TPL)	0.328	0.275	0.1851	0.301	0.2278
SM (% of TPL)	0.086	0.296	0.2849	0.096	0.3508

PL: phospholipids; TAG: triacylglycerols; TL: total lipids; TC: total cholesterol; TPL: total phospholipids; PI: phosphatidylinositol; PE: phosphotidylethanolamine; PS: phosphatidylserine; PC: phosphotidylcholine; SM: sphingomyelin.

## Discussion

Previously, compelling evidence has been provided for the effect of the K232A polymorphism of the DGAT1 gene on bovine milk fat content [16] and fatty acid composition [17]. However, no information regarding the possible effect on PL composition or MFG size has been reported. The aim of this study was, therefore, to determine the effect of both fat content and DGAT1 K232A polymorphism on milk fat composition, with a focus on PL/TAG ratio and PL composition. The main finding of our study is that DGAT1 polymorphism plays a significant role in milk fat macrostructure as reflected by PL/TAG ratio. This association of a known genetic polymorphism with MFG macrostructure provides a novel opportunity to understand how milk lipid composition is determined.

The results of the present study show that PL/TAG ratio decreases with increasing milk fat content (Table 2; Figure 1), for both DGAT1 genotypes. As the PL/TAG ratio is negatively correlated with fat globule size [13], , our data thus suggest that the size of fat globules changes with changing fat content. This is in accordance with literature on the relation between fat content and fat globule size [13], [18]. In the present study, the overall PL/TAG ratio was lower for the DGAT1 KK genotype (Table 1) suggesting that the DGAT1 KK genotype is associated with larger fat globules. This is in accordance with the finding that the DGAT1 KK genotype is also associated with higher fat content (table 1; [16]).

The association between DGAT1 genotype and PL/TAG ratio that was found in the present study may be related to differences in efficiency between the two genetic variants of the enzyme. The DGAT1 enzyme catalyzes the last step in TAG syntheses, and the K variant of the DGAT1 enzyme has been found to cause an increase in milk fat percentage, which was related to a higher V*max* compared to the A variant [16]. In addition, a common diacylglycerol (DAG) pool for both neutral and polar lipid synthesis has been suggested by a study on permeabilized hepatocytes [28]. Therefore, higher TAG-synthesis efficiency by the K variant may lead to decreased availability of DAG for polar lipid synthesis [29] which may result in sparing membrane material and, consequently, secreting MFG with lower surface area-to-volume ratio (lower PL/TAG ratio) [18]. The hypothesis that DGAT1 activity and efficiency might change the balance between PL and TAG in milk is supported by a study in which overexpression of DGAT1 in lung fibroblasts increased the utilization of the cellular pool for TAG synthesis and, in turn, decreased the concentration of all major membrane lipid constituents [30]. It is difficult to disentangle the effects of both fat content and DGAT1 genotype on PL/TAG ratio because of the highly significant interaction between DGAT1 genotype and fat content (table 2, [16]). However, when calculating the effect of DGAT1 genotype after correction for fat content, the effect of DGAT1 genotype remained significant (data not shown), suggesting an independent effect of DGAT1 genotype on PL/TAG ratio.

When the concentrations of specific phospholipids in milk were analyzed, we found that the PL content and composition (Table 1) were similar to values found previously for bovine milk [14]. We found that fat content*DGAT1 genotype interaction only tended to affect one of the major PL in milk, SM(Table 2; p = 0.086). Although not significant, the positive correlation between fat content and SM was 3 times stronger for the KK than the AA genotype (Table 2). The fact that the correlation between fat content and SM concentration did not reach significance may be attributed to the different distribution of SM between the various cellular membranes of the mammary gland epithelial cells. For example, the concentration of SM in the ER of the bovine mammary gland is four-fold lower compared with that of the plasma membrane [31]. SM concentration differed between the genotypic groups with a lower concentration for the DGAT1 AA genotype (Table 1),with a much weaker correlation between fat content and SM concentration for the AA genotype (Table 2). Cows with the DGAT1 A allele produce milk with lower palmitate and higher unsaturated fatty acids concentration [17]. We therefore hypothesize that the lower efficiency of DGAT1 A variant in incorporating palmitate into TAG will result in accumulation of palmitate in the cytoplasm and allosteric inhibition of fatty acid synthase (FAS), decreasing synthesis of its end product, palmitate. This may lead to a lower availability of palmitate in the intracellular fatty acid pool, which, in turn, may reduce *de novo* synthesis of SM that starts with a condensation step of palmitate-CoA with serine [32].

The concentration of PI was lower at higher milk fat content (Table 1) and the correlation between PI and fat content was stronger for the DGAT1 AA genotype than the KK genotype (Table 2). PS showed a significant fat content*DGAT1 interaction, with a stronger association between PS and fat content for the DGAT1 AA genotype than for the DGAT1 KK genotype (Table 2). The decrease in PI and increase in PS with increasing fat content is in agreement with our previous study [14].

PE and PC did not show a significant fat content * DGAT1 genotype interaction. The constant PC concentrations in the present study are in agreement with a previous study that showed that PC was not affected by fat globule size [14]. The fact that PE concentration was not associated with fat content was surprising since PE has previously been shown to be related to milk fat globule size [14] and since it is an important lipid in relation to membrane fusion and secretion events [33]–[35]. The MFG-secretion pathway consists of two major membrane-fusion events that potentially affect MFG size: intracellular fusion of microlipid droplets [11] and MFG secretion into the alveolar lumen. The intracellular fusion events determine the MFG diameter. A decrease in PE concentration has previously been shown during the maturation of VLDL as well as growth of intracellular lipid droplets [36], [37]. This decrease has been linked to a decrease in the lipid droplet surface curvature that results from increasing lipid droplet size. This implies a major role for PE membrane concentration in the process of intracellular fusion, and thereby possibly MFG diameter. Alternatively, metabolic pathways involving mitochondrial number and activity level, which is associated with DGAT1 genotype due to the different milk fat production levels, also influence PE concentration in the membrane, as PE is formed in the mitochondria [38]. We therefore hypothesize that the complexity of pathways regulating PE concentration, including metabolic status of the cells as well as structure-function regulation of the MFG during fusion events, make it difficult to predict the effect of fat content or DGAT1 genotype on PE.

The results of the present study have practical relevance, because there are multiple industrial implications for MFG size and lipid composition. For example, the physicochemical characteristics of cheese [28] as well as MFG coalescence and aggregation [22] have been attributed to the protein and lipid composition of small vs. large MFG. The importance of the variation in MFG lipid composition extends beyond physical properties and includes health and nutritional benefits as well. For example, the association between MFG size and fatty acid composition [29] and the relative concentration of PL [14] would be of interest to consumers’ plasma lipid profiles [4] and lipoprotein metabolism [2], [3].

In sum, there is nutritional, health and industrial interest to understand the mechanisms underlying MFG size and hence its composition.

In the present study, genotypically as well as phenotypically contrasting samples were used to further elucidate the mechanisms controlling MFGM amount and composition. The results show that DGAT1 polymorphism plays a role in determining milk total polar lipid content as well as specific lipid constituents in the polar lipid envelope of the MFG. The fact that a genetic effect was still present for some polar lipids after correcting for the effect of fat content indicates a genuine genotype effect on MFGM composition. These results provide a new direction for improving polar lipid concentration and composition in milk through selective breeding.
